# Endoscopic vitreoretinal surgery: principles, applications and new directions

**DOI:** 10.1186/s40942-019-0165-z

**Published:** 2019-06-18

**Authors:** Radwan S. Ajlan, Aarsh A. Desai, Martin A. Mainster

**Affiliations:** 1 Department of Ophthalmology, University of Kansas School of Medicine, 7400 State Line Road, Prairie Village, KS 66208-3444 USA; 2 School of Medicine, University of Missouri-Kansas City, Kansas City, MO USA

**Keywords:** Microendoscopy, Operating microscope, Ophthalmoscopy, Laser photocoagulation, Fiberoptics, Heads-up display, Gradient index lens, Hopkins rod-lens, Coherent fiberoptic, Motion parallax, Stereoendoscopy, Intraocular lens, Vitrectomy, Proliferative vitreoretinopathy, Optical coherence tomography, Surgical microscope integrated OCT, Adaptive optics

## Abstract

**Purpose:**

To analyze endoscopic vitreoretinal surgery principles, applications, challenges and potential technological advances.

**Background:**

Microendoscopic imaging permits vitreoretinal surgery for tissues that are not visible using operating microscopy ophthalmoscopy. Evolving instrumentation may overcome some limitations of current endoscopic technology.

**Analysis:**

Transfer of the fine detail in endoscopic vitreoretinal images to extraocular video cameras is constrained currently by the caliber limitations of intraocular probes in ophthalmic surgery. Gradient index and Hopkins rod lenses provide high resolution ophthalmoscopy but restrict surgical manipulation. Fiberoptic coherent image guides offer surgical maneuverability but reduce imaging resolution. Coaxial endoscopic illumination can highlight delicate vitreoretinal structures difficult to image in chandelier or endoilluminator diffuse, side-scattered lighting. Microendoscopy’s ultra-high magnification video monitor images can reveal microscopic tissue details blurred partly by ocular media aberrations in contemporary surgical microscope ophthalmoscopy, thereby providing a lower resolution, invasive alternative to confocal fundus imaging. Endoscopic surgery is particularly useful when ocular media opacities or small pupils restrict or prevent transpupillary ophthalmoscopy. It has a growing spectrum of surgical uses that include the management of proliferative vitreoretinopathy and epiretinal membranes as well as the implantation of posterior chamber intraocular lenses and electrode arrays for intraretinal stimulation in retinitis pigmentosa. Microendoscopy’s range of applications will continue to grow with technological developments that include video microchip sensors, stereoscopic visualization, chromovitrectomy, digital image enhancement and operating room heads-up displays.

**Conclusion:**

Microendoscopy is a robust platform for vitreoretinal surgery. Continuing clinical and technological innovation will help integrate it into the modern ophthalmic operating room of interconnected surgical microscopy, microendoscopy, vitrectomy machine and heads-up display instrumentation.

## Background

Operating microscope ophthalmoscopy provides stereoscopic, high resolution, widefield extraocular imaging for vitreoretinal surgery. Microendoscopic ophthalmoscopy is a parallel technology that offers non-stereoscopic, video-monitor-based, ultra-high-magnification or panoramic intraocular imaging [1, 2]. Endoscopy permits surgery for vitreoretinal disorders that are difficult or impossible to observe with transpupillary imaging because of their anatomic location or ocular media problems [3–5]. It also permits continuation of operating microscope procedures that might otherwise be interrupted by problems precluding transpupillary ophthalmoscopy [6].

The visual experience of current intraocular endoscopy is quite different from that of surgical microscopy. Endoscopy bypasses ocular media optical aberrations and pathology. It can capture images in close proximity to tissue targets, allowing it to detect minute details that would require adaptive optics [7–9] augmentation to resolve with extraocular surgical microscope ophthalmoscopy. In essence, microendoscopy is an invasive alternative to adaptive optics imaging with performance limitations that include vitreous cavity clarity and intraocular probe caliber constraints that currently restrict the resolution of intraocular optical images transferred to extraocular digital monitors.

Safe and effective use of non-stereoscopic endoscopy requires training and new competences that include judging tissue distances on 2-dimensional (2-D) video monitor images with non-stereoscopic cues such as tissue target size and structure, motion parallax, and changes in tissue color or estimated texture. Surgical and technological innovation continue to drive the evolution of endoscopic vitreoretinal surgery decades after its clinical introduction. In this analysis of ophthalmic endoscopy, we (1) summarize its basic principles, (2) review current and developing clinical applications, (3) discuss surgical challenges and complications and (4) describe emerging technology.

## Ophthalmic endoscopy principles

**Fig. 1:**
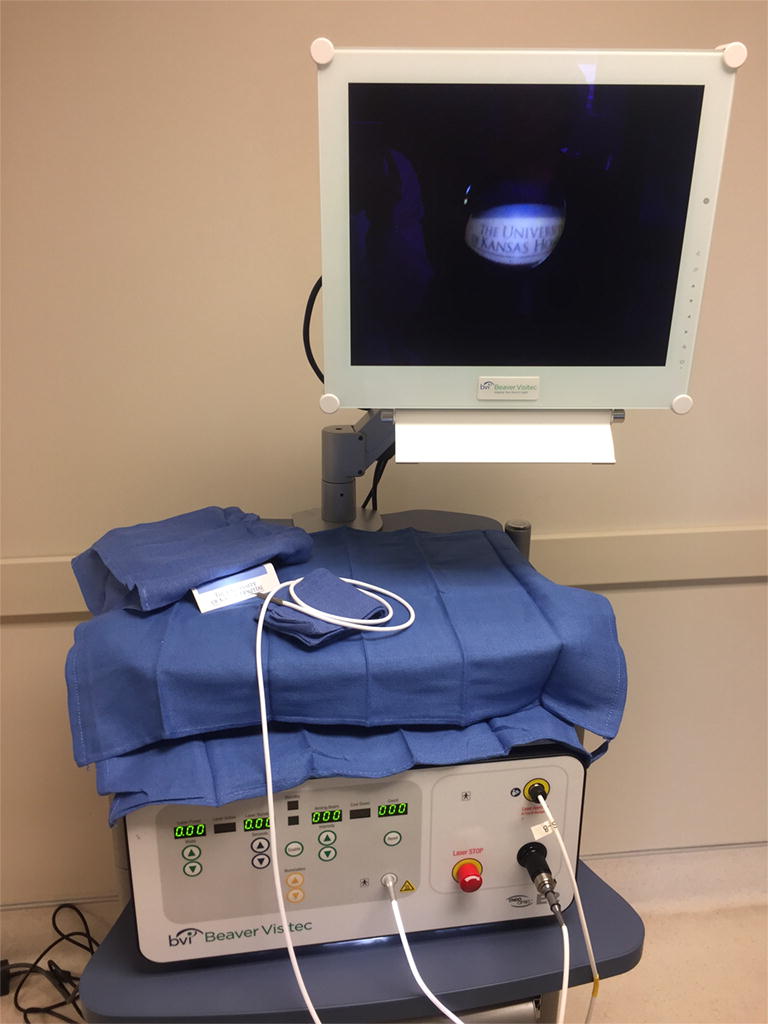
Current ophthalmic endoscopy surgical systems consist of a multifunction microendoscope and a wheeled cart housing a central console (base unit) and a two-dimensional video monitor. The base unit provides an illumination source (usually xenon), a high-resolution video camera, interfaces for microendoscope fiberoptic cabling, and connections for video output to remote monitors. It can also incorporate a photocoagulation laser source and equipment for digital image enhancement

Current ophthalmic endoscopy surgical systems consist of a multifunction microendoscope and a wheeled cart housing a central console (base unit) and a high-resolution 2-D video monitor (Fig. 1) [2, 10]. The base unit incorporates an illumination source (usually xenon), a high-resolution video camera, interfaces for microendoscope fiberoptic cabling, and connections for video output to remote monitors. It can also contain equipment for digital image processing and a laser source for photocoagulation [1, 4, 5].

### Fiberoptics: image guides, fiber bundles and laser fibers

A fiberoptic bundle consists of numerous optical fibers running parallel to each other. Each fiber has a central core surrounded by transparent cladding material that has a lower index of refraction. In widely used step-index fibers, total internal reflection limits light propagating down an optical fiber to its central core. Gradient index (GRIN) fibers constrain light to their central cores using an index of refraction that decreases radially outward from the fiber’s central axis to its periphery [11, 12].

**Fig. 2:**
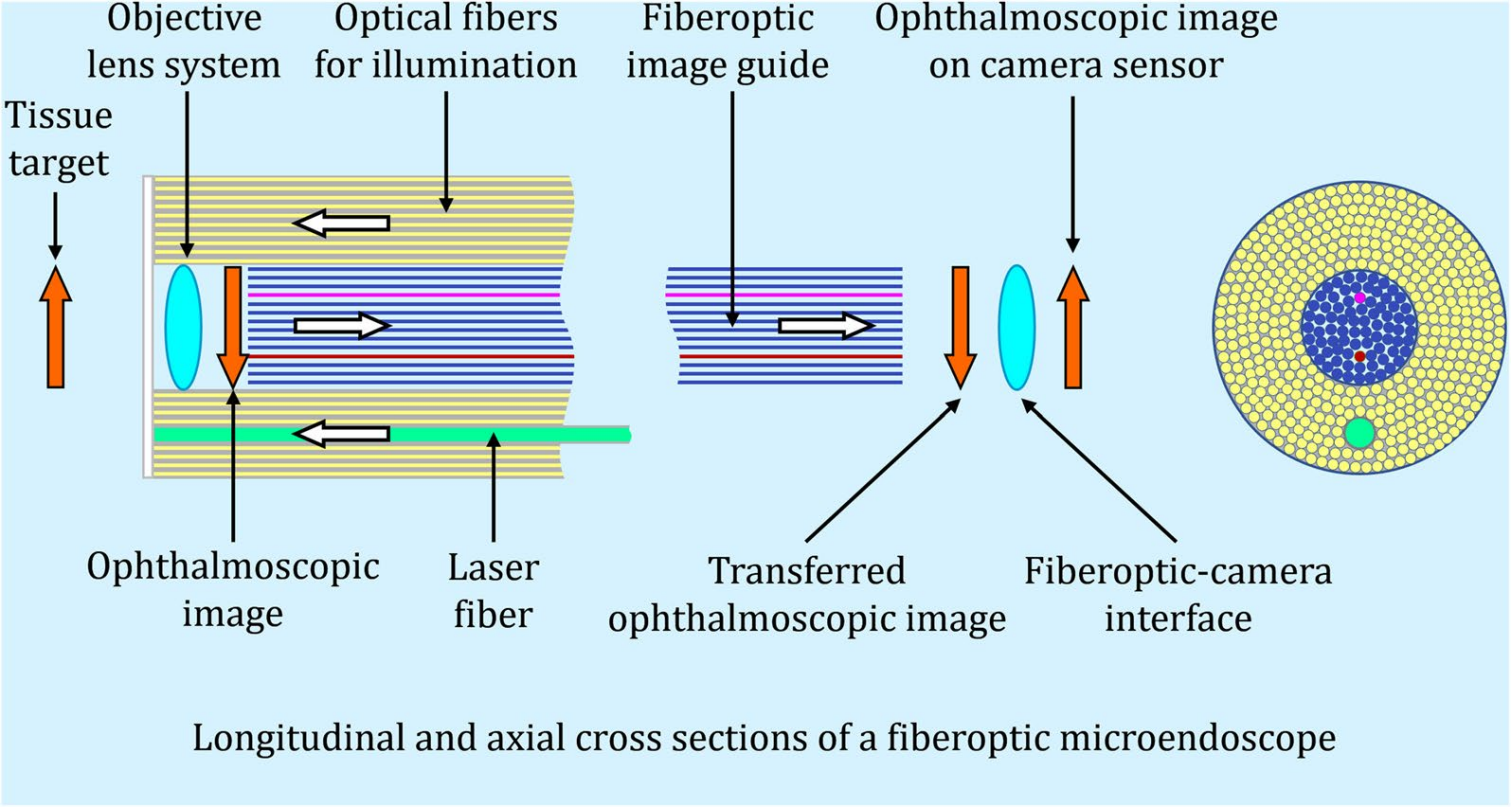
A simplified schematic diagram of an ophthalmic fiberoptic microendoscope. An objective lens system mounted at the distal (intraocular) end of an endoscope’s intraocular probe forms a high-resolution ophthalmoscopic image on the end-surface of a coherent fiberoptic bundle (image guide). The image guide relays this image to its proximal end, which is interfaced with a video camera. The camera transmits the video data to a high-resolution computer monitor. Optical fibers for illumination and laser photocoagulation extend from their central console interfaces through a handpiece to the distal end of the intraocular probe. A fiberoptic bundle is classified as coherent if the optical fibers in the bundle have same position relative to each other at the beginning and end of the bundle. Coherent bundles can serve as image guides, preserving the information in transmitted optical images. Gradient index and Hopkins rod lens microendoscopes replace fiberoptic image guides with higher resolution lens-based, image-relay systems

Fiberoptic bundles are classified as coherent if fibers have the same position relative to each other at the beginning and end of a bundle. Coherent bundles can serve as image guides, preserving the information in transmitted optical images (Fig. 2) [13]. Incoherent optical fiber bundles that don’t retain the relative positions of optical fibers are easier and less expensive to manufacture. They are useful for illumination but not transmitting optical images.

Laser photocoagulation radiation (typically 810 nm infrared radiation) is delivered by the central console’s laser source via a single, small diameter optical fiber [2]. The laser fiber is enclosed in a protective sleeve before it combines with illumination and imaging optical bundles to form a cable extending to the tip of the microendoscope’s rigid handpiece-probe. Shorter wavelength green (532 nm) or yellow (577 nm) laser sources can be used to provide more ophthalmoscopically prominent lesions at lower tissue irradiances (power/area) [14, 15].

### Microendoscope versus operating microscope imaging

Operating microscopes are used to view ophthalmoscopic images produced by contact or wide-angle non-contact lens systems [16–18]. These images are usually located in air between the lens system and the microscope. Increasing operating microscope magnification can improve the surgeon’s visualization of vitreoretinal detail in an ophthalmoscopic image but it cannot create optical information that isn’t already present in that image. Resolution in eyepiece or heads-up surgical microscope images is limited primarily by the optical performance of the ophthalmoscopic lens system and the patients’ ocular media, not by the operating microscope or video systems attached to it.

Endoscopy differs from operating microscopic ophthalmoscopy because magnifying an endoscopic image by moving the intraocular probe closer to a tissue target does create new optical information. The objective lens system mounted at the tip of the intraocular probe forms a high-resolution tissue image on the adjacent end-surface of the fiberoptic image guide (Fig. 2) [2]. That image is the intraocular equivalent of the extraocular ophthalmoscopic image viewed in operating microscopy. Microendoscopy can permit inspection of (1) minute uveovitreoretinal details beyond the resolution of contemporary, extraocular non-adaptive-optics operating microscopy and (2) inspection of uveal and very anterior vitreoretinal regions that aren’t observable in transpupillary ophthalmoscopy even with scleral depression [3–5]. Table 1 compares ophthalmoscopy using microendoscopes and surgical microscopes.

**Table 1:** Ophthalmic microendoscope and surgical microscope ophthalmoscopy

Microendoscopes	Surgical microscopes
Non-stereoscopic imaging: distances to tissue targets are judged by non-stereoscopic cues including tissue target size and structure, motion parallax and changes in tissue color or estimated texture	Full stereoscopic imaging: precision of depth judgement depends on the surgeon’s stereoacuity
Larger or smaller fields of view and laser spot sizes are produced by moving the probe tip farther from or nearer to tissue targets, respectively; lens-based microendoscopes currently provide higher resolution images than fiberoptic devices	Non-contact ophthalmoscopic lens systems offer wide-field, high resolution imaging; contact systems can increase magnification and resolution in part by eliminating corneal aberration and reflection
Magnification is increased by moving the intraocular probe closer to a tissue target, creating new detail in intraocular optical images and their digital representations on a video monitor (the surgeon increases both ophthalmoscopic image magnification and resolution)	Increasing operating microscope magnification can improve visualization of information in an ophthalmoscopic image but it cannot create detail that wasn’t already present in the image (the surgeon magnifies their view of the ophthalmoscopic image without increasing its resolution)
Fine uveovitreoretinal detail undetectable with extraocular ophthalmoscopy can be imaged by moving the microendoscope probe close to a tissue target. Resolution in this ultra-high magnification optical image is reduced when it’s displayed on a digital monitor dependent on multiple factors including vitreous cavity clarity and the number of optical fibers in a fiberoptic image guide (Fig. 2)	Visible vitreoretinal tissue detail is limited by optical aberrations in ocular media and ophthalmoscopic lenses and by ocular pathology that interferes with transpupillary ophthalmoscopy. Adaptive optics technology that could correct for ocular media aberrations is not available in contemporary operating microscopes
Images can be viewed on the microendoscope’s dedicated small screen display or larger high definition operating room monitors	Images can be viewed through the microscope’s eyepieces. Alternatively, video cameras can transfer bilateral microscope images to heads-up devices such as high definition, three-dimensional monitors

### Fiberoptic versus non-fiberoptic microendoscopes

In a contemporary ophthalmic fiberoptic microendoscope, optical fibers for illumination, imaging and optional laser photocoagulation extend from their console interfaces through a handpiece to the distal end of an intraocular probe (Fig. 2). The fiberoptic image guide relays the initially high-resolution optical image on its distal intraocular tip to its proximal end, which is interfaced with a digital video camera (Fig. 2). Resolution lost in this fiberoptic transmission depends largely on the number of optical fibers in the image guide (individual optical fibers correspond to screen pixels [2]).

Outside the endoscope’s rigid metallic handpiece-probe, fiberoptic bundles are encased in a mechanically-protective sleeve. Fibers in these cables can break if the cables are bent excessively or otherwise misused. Each broken optical fiber in the image guide eliminates a video pixel, producing a black spot on the monitor that reduces the endoscope’s resolution and its clinical lifetime.

Smaller caliber microendoscope probes are currently produced by reducing the number of image guide optical fibers. Reducing that number decreases a microendoscope’s (1) optical resolution because fewer pixels of ophthalmoscopic image information are transferred to the video camera and (2) field of view because the reduced diameter image guide captures a smaller area of the ophthalmoscopic image for a particular objective lens system (the intraocular end of the image guide bundle serves as an optical field stop).

Straight, curved and bent-tip rigid microendoscope probes have been developed [2]. Probes are currently available in 19-, 20-, 23- and 25-gauge sizes [4, 5, 19]. Current smaller caliber microendoscopes have fewer image guide optical fibers, lower resolution and smaller imaging fields. Intraocular depth of field ranges from roughly 0.75–40 mm, permitting high magnification when the endoscope probe is adjacent to tissues and a panoramic intraocular view when it’s close to the sclerotomy site [2]. Wider perspectives are especially helpful for instrument localization in bimanual procedures.

Hopkins rod-lenses and GRIN lenses currently retain more resolution than fiberoptic bundles in intraocular images relayed to extraocular cameras [2, 4]. Hopkins rod-lens endoscopes use small diameter cylindrical lenses with large length-to-diameter ratios to transfer the ophthalmoscopic image from the distal (intraocular) tip of the probe to the proximal end of the handpiece [20]. GRIN lens endoscopes replace conventional objective and relay lenses with small diameter GRIN lenses. A conventional lens refracts light using the curved surfaces of homogeneous optical material that has a single index of refraction. GRIN lenses refract light using inhomogeneous optical material with refractive indices that vary radially, longitudinally or spherically [11, 12]. These lenses are often fabricated with flat ends to facilitate their use in multiple-lens systems and bonding to fiberoptic components.

Current ophthalmic GRIN endoscope probe-handpiece units are attached directly to a video camera to exploit their high resolution. This endoscope-camera configuration is less maneuverable in surgery than a fiberoptic microendoscope. Additionally, GRIN systems are relatively fragile and their non-autoclavable camera must be draped separately for surgery [2, 21]. In essence, current GRIN microendoscopes achieve higher resolution by sacrificing the surgical convenience and current lower cost of fiberoptic devices. Table 2 compares the features of fiberoptic and gradient index lens ophthalmic microendoscopes.

**Table 2:** Features of fiberoptic and gradient index lens ophthalmic microendoscopes

Fiberoptic microendoscopes	Gradient index (GRIN) lens microendoscopes
A fiberoptic image guide bundle extends from the distal tip of the rigid intraocular probe through the probe and its attached handpiece to the central console	A video camera or viewing eyepiece attaches directly to the comparatively fragile GRIN lens handpiece-intraocular probe
Readily maneuverable in surgery	Less maneuverable in surgery because of the direct attachment of the video camera and/or viewing eyepiece to the handpiece-probe unit
The entire fiberoptic microendoscope can be autoclaved	The handpiece-GRIN lens assembly can be autoclaved but not its video camera which must be draped separately for operating room use
Image resolution is limited by the number of optical fibers in the image guide: smaller caliber microendoscopes have fewer fibers, lower resolution and smaller imaging fields	GRIN lenses in the probe-handpiece relay the intraocular ophthalmoscopic image directly to the video camera, retaining much of its high spatial frequency, fine detail information
Coaxial illumination may be supplemented with additional chandelier or endoilluminator lighting	Coaxial illumination may be supplemented with additional chandelier or endoilluminator lighting

### Microendoscope illumination

Endoilluminators and chandelier devices are used in vitreoretinal operating microscope procedures to illuminate surgical fields [22], eliminating the potentially dazzling ocular media light reflexes of extraocular light sources. Surgical microscopes use accessory non-contact or contact lens systems to form extraocular ophthalmoscopic images from tissues that are obliquely illuminated by these devices. Conversely, endoscopes illuminate tissue targets coaxially, using an objective lens system to form an intraocular ophthalmoscopic image with light scattered directly back at them from tissue targets. Tissues can be illuminated at different viewing angles in microendoscopy, optimizing visualization of subtle vitreous structures poorly identified in non-coaxial illumination or hidden by the instrument shadows or glare it causes [4, 10, 23].

## Clinical applications

Microendoscopic ophthalmoscopy can circumvent ocular media obscuration, identify tiny uveovitreoretinal abnormalities and image intraocular areas difficult or impossible to view with contemporary operating microscope ophthalmoscopy. Numerous procedures exploit these capabilities.

### Posterior chamber intraocular lenses

**Fig. 3:**
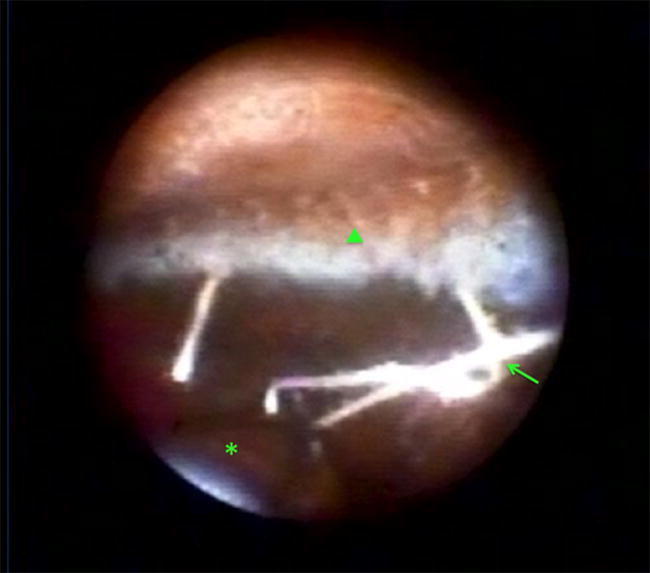
Endoscopic view showing the inverted orientation of a scleral-fixated aspheric intraocular lens (asterisk), overlapping polytetrafluoroethylene monofilament (GORE-TEX^®^) sutures (arrow), and ciliary body processes (arrowhead)

Intraocular lens (IOL) implantation is an important part of some pars plana vitrectomy (PPV) procedures. Sutured, sulcus-fixated posterior chamber IOLs offer effective post-operative visual rehabilitation in adult and pediatric patients. Endoscopy can assure proper sulcus haptic placement and IOL centration (Fig. 3) but increases procedure duration [24].

### Keratoprostheses

Endoscopic evaluation is helpful before keratoprosthesis surgery if a patient’s visual potential or vitreoretinal condition is uncertain [25, 26]. Endoscopic cyclophotocoagulation can be performed after implantation [27]. In eyes with an opaque cornea following ocular trauma, endoscopic surgery can allow earlier diagnosis and treatment of occult retinal tears and detachment, shorter operation times and fewer procedures than operating microscopy with a temporary keratoprosthesis [28, 29]. Earlier treatment is particularly helpful in pediatric patients where delays can cause severe proliferative vitreoretinopathy (PVR) and amblyopia [30].

### Retinal prostheses

The Argus II implant is approved by the U.S. Food and Drug Administration for use in patients with retinitis pigmentosa over 25 years of age whose prior useful vision has declined to minimal or no light perception [31, 32]. The implant’s 60 electrode array is inserted through a 5.2 mm sclerotomy into the vitreous cavity after vitrectomy. The array is centered over the macula and tacked to its inner retinal surface [31, 32]. A spectacle-mounted camera transmits video data to the electrodes to stimulate inner retinal cells still functioning after photoreceptor degeneration. Endoscopy and intraoperative OCT can reduce retinal damage and other intraoperative complications by helping guide sclerotomy as well as electrode array positioning and tacking [31, 33].

### Subretinal hemorrhages in macular degeneration

Large subretinal hemorrhages from neovascular age-related macular degeneration cause profound, acute vision loss. Endoscopic surgery can be used to evacuate the subretinal blood and remove contributing fibrovascular retinal pigment epithelium without the need for large retinotomies. In a small case series, visual acuity improved in three of five patients and PVR did not occur within a 12-month follow-up period [34].

### Intraocular foreign bodies

Ocular trauma can compromise media clarity thereby impairing surgical microscope ophthalmoscopy. Endoscopy bypasses this problem, permitting prompt identification and management of vitreoretinal problems and intraocular foreign bodies [29, 35–37]. Endoscopy can shorten trauma-to-surgery and surgical time. Its outcomes are similar to those of operating microscopy with a temporary keratoprosthesis but avoids some of the logistical issues associated with temporary implant use [29].

### Internal limited membrane peeling

Endoscopy was used to repair a macular hole-related retinal detachment in a patient with severe keratoleukoma and pathologic myopia. Epiretinal membrane and indocyanine green-assisted internal limiting membrane peeling were monitored with a 23-gauge microendoscope. Silicone oil was removed 6 months post-operatively and the retina remained attached 12 months after surgery [38].

### Scleral depression

Scleral depression in operating microscope procedures permits ciliary body and peripheral retinal visualization. Alternatively, endoscopy doesn’t require scleral depression which hypothetically might contribute to postoperative inflammation and proliferation [5, 39]. Endoscopy without scleral depression identified retinal tears or holes in 19 of 20 consecutive patients whose breaks were undetectable preoperatively by indirect ophthalmoscopy with scleral depression [39]. The retina was reattached in all 20 patients after a single operation.

### Proliferative vitreoretinopathy

**Fig. 4:**
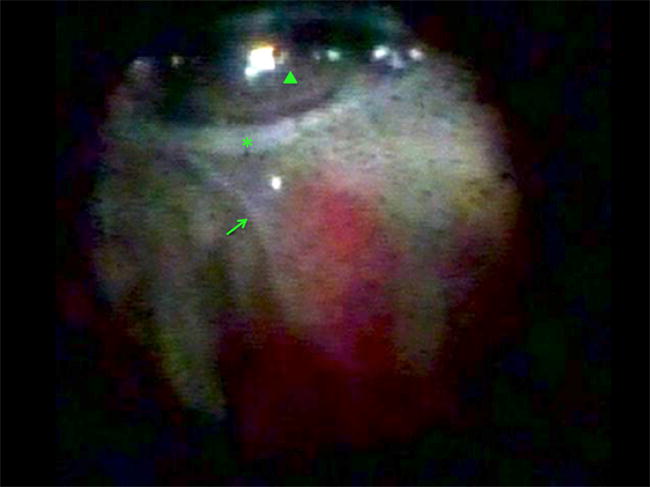
Endoscopic view in a patient with chronic retinal detachment and hypotony showing a tractional retinal detachment (arrow) connected to an epiciliary membrane (asterisk). An air bubble is located superiorly (arrowhead)

**Fig. 5:**
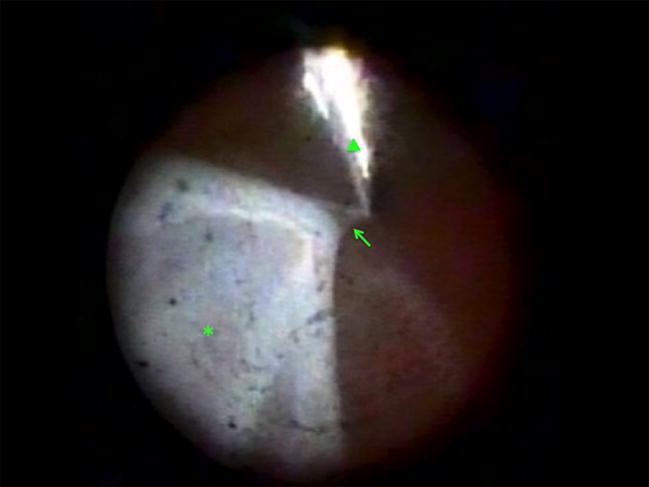
Endoscopic view during a preretinal proliferative vitreoretinopathy (arrow) peel with forceps (arrowhead) in a patient with a retinal detachment (asterisk) and a corneal scar that prevented effective operating microscope ophthalmoscopy

Anterior PVR can cause ciliary body fibrosis and detachment leading to hypotony and phthisis (Fig. 4) [40, 41]. Endoscopy is a valuable tool for assessing ciliary body status and determining when to perform silicone oil refilling [40, 42]. Endoscopic vitrectomy to remove PVR from the ciliary body can be helpful in some cases of chronic hypotony, particularly in younger patients or those with fewer previous procedures (Fig. 5) [41].

**Fig. 6:**
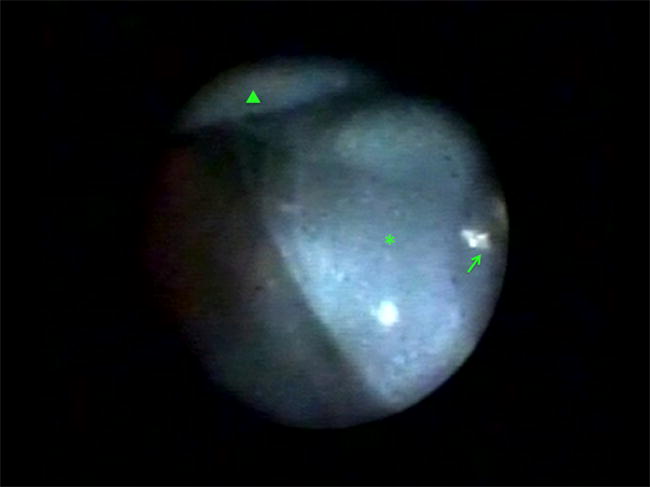
Endoscopic view during pneumatic dissection of the anterior hyaloid membrane in phakic patient. The needle tip is located in the canal of Petit (arrow), with the anterior hyaloid membrane being dissected (asterisk) from the crystalline lens (arrowhead)

In endoscopy-assisted anterior hyaloid membrane dissection, a 30-gauge needle can be placed in the canal of Petit and filtered air injected to dissect the anterior hyaloid membrane from the lens capsule (Fig. 6). The vitrector can then be used to remove the anterior hyaloid and vitreous [43, 44].

Operating microscope ophthalmoscopy is hampered by reflections from air-fluid interfaces. Endoscopic surgery for posterior PPV with a partial air-fluid exchange permits posterior membrane peeling under water while air in the anterior part of the vitreous cavity flattens the detached retina [45]. This technique benefits from the increased maneuverability in patient head-positioning of endoscopy compared to surgical microscopy.

Surgical microscope visualization of posterior PVR is better under water that than air but endoscopy can provide improved viewing in air (“atmospheric endoscopic technique”) by avoiding anterior segment image degradation [46, 47]. Subretinal proliferation in severe posterior PVR can be removed without the need for retinotomies by inserting a microendoscope beneath detached retina [47].

## Surgical challenges and complications

Heads-up display of stereoscopic operating microscope fields on a three-dimensional (3-D) monitor can reduce the cervical strain of eyepiece usage [48]. Monitor-based endoscopic surgery has the same benefit but operating on the 2-D representations of 3-D structures increases surgical visual and cognitive workloads [49]. New skills that must be mastered include remaining oriented in a 2-D intraocular field, adjusting illumination for probe-tissue distances, staying in the center of the endoscope’s image circle when manipulating tissue, and assessing distances from vital intraocular structures using non-stereoscopic cues such as motion parallax, estimated structure size or perceived tissue texture.

Inadequate endoscope illumination can limit target tissue image quality. Chandelier lighting systems can be helpful in such situations by diffusely illuminating a larger intraocular volume. Their disadvantages include instrument shadows and reflections that can impair tissue visualization [23]. An additional endoilluminator can be useful if retinal phototoxicity risks and glare are minimized.

Tissue visualization can be compromised by vitreous hemorrhage, especially in cases of proliferative diabetic retinopathy or PVR. A fluid-air exchange or use of a liquid perfluorocarbon (e.g., perfluoro-*n*-octane) may help resolve imaging issues. Visualization can also be impaired by endoscope probe tip fogging after a fluid-air exchange. Defogging can be accomplished by repositioning the tip in fluid or retracting and cleaning it.

Some endoscopically visible tissues may be difficult to reach with a vitrectomy instrument because of cannula positioning, imaging limitations or a patient’s crystalline or intraocular lens. Exchanging the port locations of the microendoscope with the vitrectomy probe or infusion line can help solve access problems in these situations.

Endoscopic surgery complications that are infrequent for rhegmatogenous retinal detachment repair occur more commonly in advanced disorders [4]. In one series of 12 patients treated for severe endophthalmitis, iatrogenic retinal tears occurred in 4 eyes, including a giant flap tear related to vitreous base clean-up in one eye [50].

**Fig. 7:**
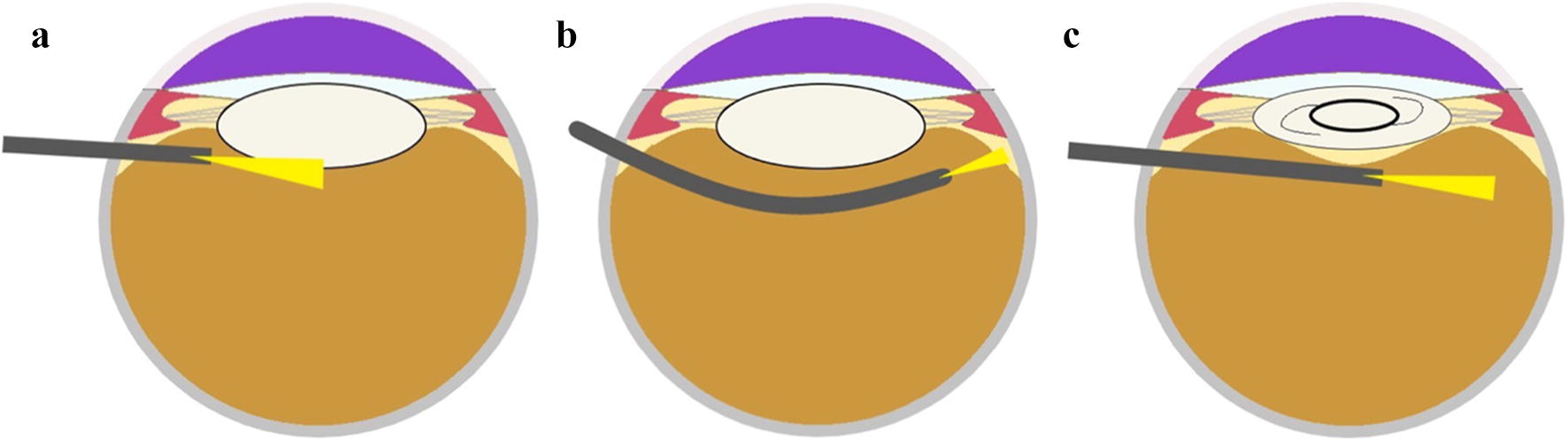
Schematic diagram of ophthalmic endoscopy. **a** Shows a straight endoscopy probe in proximity to the crystalline lens to emphasize that the risk of iatrogenic cataract can be reduced if the surgeon avoids passing instrumentation behind the posterior pole of the crystalline lens. **b** Shows how a curved endoscope probe can facilitate far periphery examination without crystalline lens injury. **c** Illustrates that a straight endoscopy probe can be used to cross behind the posterior pole of an artificial intraocular lens

Iatrogenic secondary cataract can be a distressing problem. Secondary cataract occurs in 6–20% of pediatric patients undergoing lens-sparing operating microscope vitreous surgery, mostly due to crystalline lens-retina approximation or instrument-crystalline lens touch [51, 52]. Endoscopy permits a unique view of instrumentation positioning relative to the crystalline lens. Maneuvering to avoid contact with the posterior lens surface can be challenging. In general, the risk of iatrogenic cataract can be minimized if the surgeon avoids passing instrumentation behind the posterior pole of the crystalline lens (Fig. 7) [52].

## Emerging technology

Endoscopic probe diameters are restricted by the caliber limitations of intraocular surgery. Some of the capabilities non-ophthalmic endoscopy may be achievable with emerging technology or the sequential use of specialized microendoscopes and intraocular probes. Innovation in microendoscopy faces the same conundrum in ophthalmology as it does in other surgical areas: demand requires availability which requires demand to justify development, manufacturing and distribution costs.

### Stereoscopic microendoscopy

Stereoscopic endoscopy has been available but not uniformly accepted in disciplines including urology, gynecology and otolaryngology [49, 53, 54]. The potential advantages of 3-D vs. 2-D endoscopy include shorter learning curves for surgery, increased recognition of tissue texture and curvature, fewer surgical errors from visual misperception, decreased operating times, more precise depth judgment and decreased perceived workload [49, 54–56]. A prototype 18-gauge stereoscopic ophthalmic microendoscope was reported decades ago [57].

New technologies that potentially facilitate small caliber 3-D endoscopy include replacing optical fiber image guides with video microchips mounted on the distal end of endoscope probes (“chip-on-the-tip” sensor elements rather than optical cores correspond to video monitor pixels) [58]. Dual-channel systems that operate similar to the human stereopsis and single channel devices that use a lens array overlying a single microchip (“insect eye” or “plenoptic” camera) are both used in non-ophthalmic stereoendoscopic surgery [59, 60]. Evolving single-channel stereoendoscopic technology includes rotating deflector and single-lens, dual-aperture devices [61, 62].

Stereoscopic endoscopy has technical challenges that include realistically representing depth information despite the huge disparity between a surgeon’s interocular distance and the effective separation distance of left and right image channels at a stereoendoscope’s tip (“gigantism”) [63–65]. Equipment-related and operator stereopsis-dependent side effects reported by some users of non-ophthalmic 3-D endoscopic systems include dizziness, headache and spatial disorientation [49, 63, 66, 67].

### Reflectance and fluorescence

Standard microendoscopic light sources provide broad-spectrum white light. Illumination is reflected back into a coaxial imaging channel (fiberoptic bundle, grin lens or video microchip). Specular reflections from insubstantial vitreous structures can help identify tissues that are largely invisible in the side-scattered light of operating microscope intraocular illuminators [4, 10].

Microendoscope illumination and imaging channels can be color-filtered to permit endoscopic monochromatic ophthalmoscopy [68] or fluorescein angiography [69]. Indocyanine green (ICG) angiography and autofluorescence imaging could be performed with appropriate filter sets. ICG, trypan blue and other vital dyes have been used to stain structures in vitreoretinal operating microscope procedures [70, 71]. They could also be used in endoscopic chromovitrectomy.

### Digital image processing

Vitreoretinal endoscopic images can be computer-enhanced in real-time before they are displayed on a video monitor. Image size, contrast and color balance adjustment have been available [5]. Additional computer techniques for real-time enhancement of surgical images include spatial frequency modulation (filtering) [72, 73], tissue topology and texture recognition [74], adaptive illumination adjustment [75] and image stabilization [76].

### Robotic microscopic and endoscopic surgery

Robotic endoscopic surgery is a mature discipline in urology, gynecology and other non-ophthalmic medical specialties [77]. Early efforts are underway to develop specialized robotic devices for ophthalmic surgery or use mature non-ophthalmic technology in ophthalmic applications [78, 79].

### Optical fiber flexibility and prisms

Ophthalmic microendoscopes currently use rigid straight or curved intraocular probes for vitreoretinal or more anterior surgery, respectively [4]. Flexible, sterilizable endoscopes are used in other surgical disciplines where larger diameter endoscopes can accommodate channels for illumination, imaging, instrumentation and tip-bending control wires [80, 81]. Single-use illumination and laser probes are available for vitrectomy using fiberoptic bundles that curve when extended from a rigid intraocular housing [82].

Small diameter coherent image guides have been developed with enough flexibility to bend around a small coin without fiber breakage. The two ends of fiberoptic bundles are fused so that individual optical fibers maintain the same relative position at both ends of the bundle, thereby providing the coherence needed for image transmission. Outer cladding material elsewhere is removed (acid “leached”) to separate fibers and produce a highly flexible fiber bundle [13].

Improved flexibility in a coherent imaging bundle can increase microendoscope longevity. Flexible-tip, single-use fiberoptic image guides are currently too expensive for surgical use. Flexible-tip, multi-use multifunction probes with instrumentation channels and guide wires aren’t feasible for ophthalmic endoscopy because of intraocular probe caliber limitations. An alternative way to extend microendoscopic field of view is to incorporate a prism into the objective lens system at the distal end of a rigid, straight intraocular probe. This technology has been developed for GRIN microendoscope probes [83].

### Augmented imaging

Large screen, ultra-high-definition 3-D monitors can be used as a heads-up display for surgical microscopes [48, 84, 85]. Ample screen space is available for additional intraoperative data including (1) real-time optical coherence tomography (OCT) or microendoscope imaging and (2) technical details such as intraocular and infusion pressures [48, 85]. Supplementary data can be available as needed but otherwise not distracting. Protocols driven by physician, government and/or industrial groups may eventually compel manufacturers to make data streams from different operating room imaging sources compatible as for example was done for medical images with the Digital Imaging and Communications in Medicine (DICOM) standard.

Microendoscopy is currently used primarily as an independent surgical platform. Periodic switching between viewing a small endoscopy monitor and operating microscope oculars is helpful for staying oriented in the surgical field and positioning endoscopic instrumentation. Large, multiple or split-screen, ultra-high-definition monitors can eliminate this task by displaying both information streams simultaneously and also improving endoscope display quality [85].

Operating microscope-integrated OCT (MIOCT) systems can currently display 2-D or 3-D OCT data in microscope oculars [86–88]. Supplemental intraoperative OCT data could be made available during microendoscope procedures from sources including: (1) MIOCT systems, (2) independent intraocular OCT probes or (3) very small diameter OCT probes integrated into microendoscopes. Intraocular OCT probes are in the developmental stage [89].

## Summary

Microendoscopy permits effective surgery on intraocular structures impossible to view with transpupillary ophthalmoscopy because of their location or ocular media opacification. It’s range of vitreoretinal applications continues to expand. Developing technology promises further expansion of those capabilities as well as more effective integration of ophthalmic operating room microscope, microendoscope, vitrectomy machine and heads-up display instrumentation.

## Abbreviations

2-D: two dimensional
3-D: three dimensional
GRIN: gradient index
IOL: intraocular lens
PPV: pars plana vitrectomy
PVR: proliferative vitreoretinopathy
OCT: optical coherence tomography
ICG: indocyanine green
DICOM: Digital Imaging and Communications in Medicine
MIOCT: surgical microscope integrated OCT
